# Supplementary material for: Determining the Potential of DNA Damage Response (DDR) Inhibitors in Cervical Cancer Therapy
Source: Cancers (Basel). 2022 Sep 1;14(17):4288. doi: 10.3390/cancers14174288 (PMC9454916; doi:10.3390/cancers14174288)
Supplement: Supplementary file 1 [file cancers-14-04288-s001.zip › cancers-1859889-supplementary.pdf]

cancers

MDPI

Supplementary

Determining the potential of DNA damage response (DDR) inhibitors in cervical cancer therapy

Santu Saha [1,†], Stuart Rundle [2,†], Ioannis C. Kotsopoulos [3], Jacob Begbie [4], Rachel Howarth [1], Isabel Y. Pappworth [5], Asima Mukhopadhyay [6], Ali Kucukmetin [7], Kevin J. Marchbank [5] and Nicola Curtin [1,*]

1 Translational and Clinical Research Institute, Faculty of Medical Sciences, Newcastle University, Framlington Place, Newcastle upon Tyne NE2 4HH, UK
2 The Northern Gynaecological Oncology Centre (NGOC), Queen Elizabeth Hospital, Gateshead NE9 6SX, UK
3 University College London Hospitals NHS Foundation Trust, 250 Euston Rd, London NW1 2PG, UK
4 Addenbrooke's Hospital, Cambridge, CB2 0QQ, UK
5 Translational and Clinical Research Institute, National Renal Complement Therapeutics Centre, Newcastle University, Newcastle upon Tyne NE2 4HH, UK
6 Kolkata Gynecological Oncology Trials and Translational Research Group, Chittaranjan National Cancer Institute, Kolkata 700026, India and Department of Gynaecological Oncology, James Cook University Hospital, Middlesbrough TS4 3BW, UK and Newcastle University, Newcastle upon Tyne NE2 4HH, UK
7 Northern Gynaecological Oncology Centre, Queen Elizabeth Hospital, Gateshead NE9 6SX, UK and Newcastle University, Translational and Clinical Research Institute, Newcastle upon Tyne NE2 4HH, UK
* Correspondence: nicola.curtin@newcastle.ac.uk
† These authors contributed equally to this work.

List of supplementary materials

1. Supplementary Figure S1. Determining maximum tolerated dose of cisplatin (Materials and Methods, Section 2.7).

2. Supplementary Table S1. Histopathological classification, HPV status and TP53/RB1 status of the panel of 6 cervical cancer cell lines (Results, Section 3.1).

3. Supplementary Table S2. Growth rate and cloning efficiency of cervical cancer cell lines (Results, Section 3.1).

4. Supplementary Figure S2. The relative expression of the DDR proteins as given in main Figure 1A.

5. Supplementary Figure S3. Representative immunoblot images of the PARP activity among the cervical cancer cell lines (Results, Section 3.1).

6. Supplementary Table S3. PARP activity among the cervical cancer cell lines (Results, Section 3.1).

7. Supplementary Figure S4. DDR pathways activation and inactivation as given in main Figure 2C and 2D.

8. **Supplementary Figure S5.** Optimisation of cisplatin concentration for PARP activation.

9. **Supplementary Table S4.** The cytotoxicity of the DDR inhibitors alone in the cervical cancer cell lines as given in main Figure 3A-D.

10. **Supplementary Figure S6.** Sensitivity to IR and cisplatin to the cervical cancer cells (Results, Section 3.2).

11. **Supplementary Table S5.** Radiopotentiation of the DDR inhibitors in the cervical cancer cell lines as given in main Figure 3E-H.

12. **Supplementary Table S6.** Cisplatin sensitisation of the DDR inhibitors in the cervical cancer cell lines as given in main Figure 3I-L.

13. **Supplementary Figure S7.** Chemo-radiosensitisation by rucaparib in cervical cancer cells (Results, Section 3.2).

14. **Supplementary Figure S8. Cell cycle histogram profiles of cervical cancer cell lines treated with vehicle control (0.5% DMSO), or 3 μM cisplatin (Cis) +/- 1 μM VE-821 (VE), 50 nM PF-477736 (PF) or 100 nM MK-1775 (MK) as given in Figure 4A and in Supplementary Table S7.**

15. **Supplementary Table S7. Cell cycle profiles of cervical cancer cell lines treated with vehicle control (0.5% DMSO), or 3 μM cisplatin (Cis) +/- 1 μM VE-821 (VE), 50 nM PF-477736 (PF) or 100 nM MK-1775 (MK) as given in Figure 4A** (Results, Section 3.3).

16. **Supplementary Figure S9.** Histology of kidney sections (Results, Section 3.4).

17. **Supplementary Figure S10.** Determinants of sensitivity to the DDR inhibitors (Discussion, Section 4).

18. **Supplementary references**

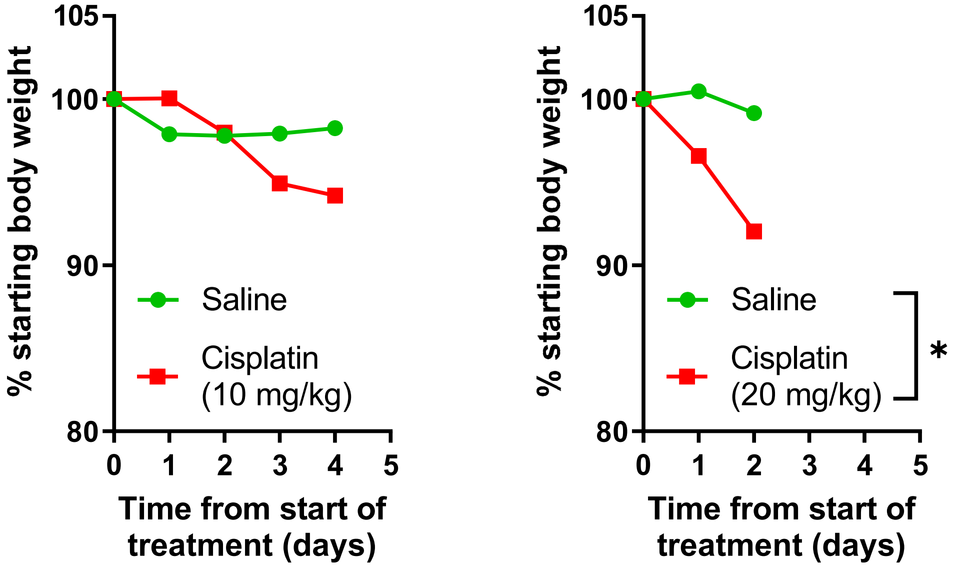

Supplementary Figure S1. Determining maximum tolerated dose of cisplatin to establish cisplatin-induced acute kidney injury model in wild type CD1 mice: For establishing the cisplatin-induced acute kidney injury model 2 different doses of cisplatin (10 mg/kg and 20 mg/kg) were tested. For the 5 days acute kidney injury model, cisplatin was injected on day 1 through the intraperitoneal (i.p.) injections (n = 3 per group). Safety and tolerability of cisplatin was measured by the parameters of body weight, body posture (e.g. hunching), movement/hypokinesia, grooming ability/piloerection and eye colour. Mice were checked for signs of any ill health 3-5 times daily. The graphs represent body weights of the mice (n = 3 mice per group). Unpaired T test, *, **, ***, **** are $p < 0.05$, 0.01, 0.001 and 0.0001, respectively.

Cell Line	Histopathological sub-type and tumour site.	HPV status	Pathogenic mutations
HeLa	Primary cervical adenocarcinoma	HPV 18+	-
SiHa	Primary cervical squamous cell carcinoma	HPV 16+	-
C33A	Primary cervical carcinoma	-	TP53/RB1
CaSki	Cervical carcinoma from metastatic site: small intestine	HPV 16+ HPV 18+	-
ME-180	Cervical squamous cell carcinoma from metastatic site: omentum	HPV 68+	-
HT-3	Cervical carcinoma from metastatic site: lymph node	-	TP53/RB1

Supplementary Table S1. Histopathological classification, HPV status and TP53/RB1 status of the panel of 6 cervical cancer cell lines started using in the study.

Cell Line	Cell doubling time hours ± SEM (n)	Observed cloning efficiency % ± SEM (n)
HeLa	44 ± 3 (3)	47 ± 2 (3)
SiHa	59 ± 3 (3)	39 ± 5 (3)
C33A	46 ± 5 (3)	73 ± 13 (3)
CaSki	46 ± 3 (3)	24 ± 7 (3)
ME-180	30, 33 (2)	51 ± 7 (3)
HT-3	37 ± 2 (3)	22 ±1 (3)

Supplementary Table S2. Growth rate and cloning efficiency of cervical cancer cell lines further studied to include selected cell lines for further study: Growth rate was determined as the doubling time of the cells and the method is described below. Cloning efficiency of the cells were determined as described in the main text section 2.3. Data are mean and SEM of the mean of three biological replicates. For ME-180 cells, the mean doubling time of two biological replicates are given.

Method to determine cell growth: Exponentially growing cells were seeded into five rows of six 96-well plates at a different seeding/row ranging from 6.75 x10^3 cells/well to 1.0 x 10^5 cells/well in 100 µl medium and incubated. One plate was fixed (methanol: acetic acid 3:1) at daily intervals, washed, dried and stored until the final plate was fixed then all plates were stained witn 20 µl 0.04% w/v sulphurhodamine B for 1 h, washed and dried prior to the addition of 100 µl of 10 mM Tris-base (pH 10.5). Absorbance at wavelength 510 nM was measured using a FLUOstar® Omega microplate reader. Absorbance vs time graphs were constructed using GraphPad Prism SanDiego, CA, USA) software which was used to calculate cell doubling time.

Among the 6 cell lines, we considered to take only 4 cell lines forward for rest of the main experiments. These cells are belonged to high-risk HPV positive: HeLa (HPV 18+), SiHa (HPV 16+) and CaSki (HPV16+ and HPV18+) and HPV negative but harbour TP53 +/- RB1 mutations (C33A). Clonogenic efficiency of HT3 was lowest and Me180 contain HPV 68, which is the rarest HPV type found in the cervical cancer patients. Therefore, these two cell lines were excluded.

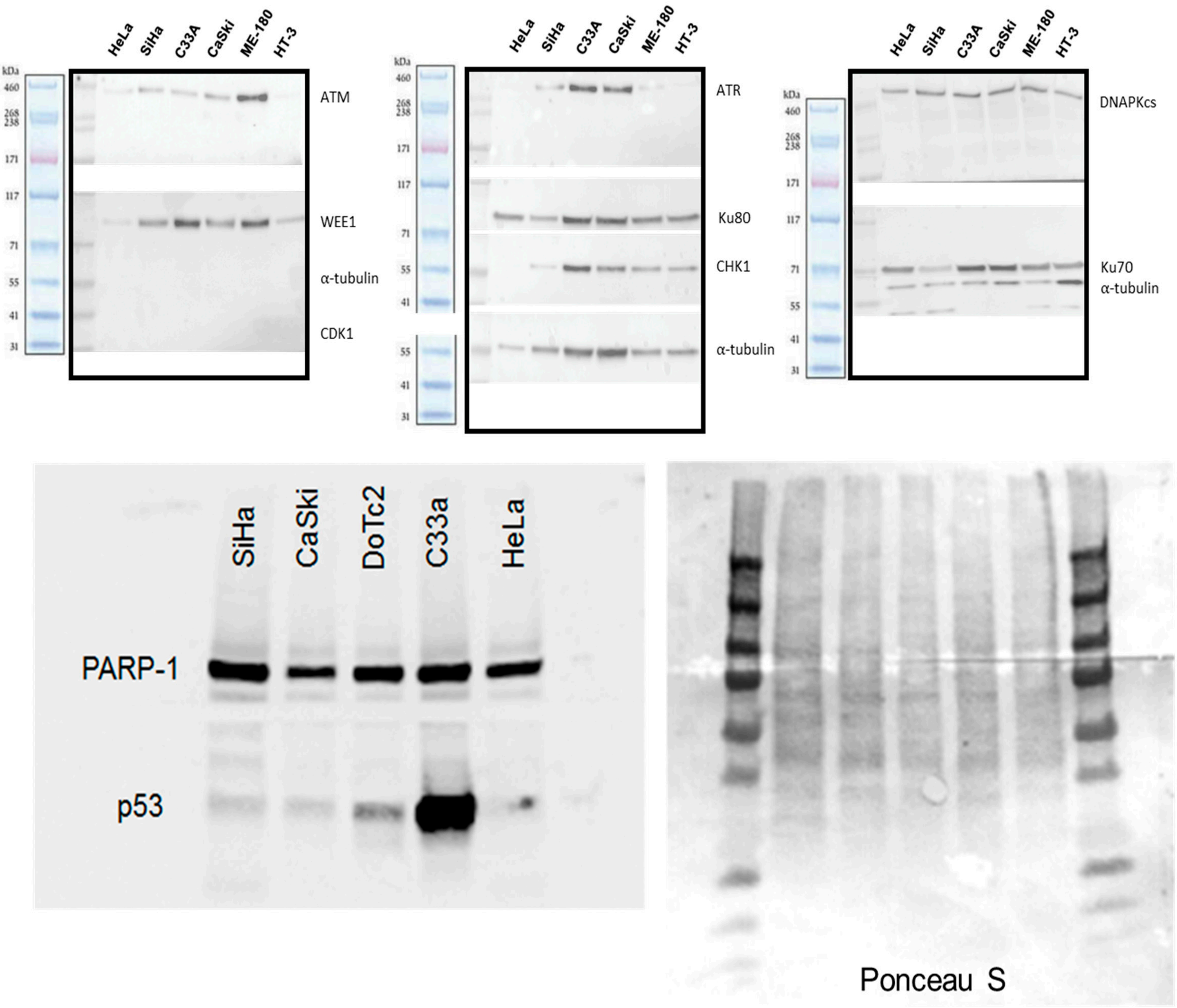

Supplementary Figure S2. The relative expression of the DDR proteins as given in main Figure 1A: The expression of the S and G2-M cell cycle checkpoint kinases (ATR, CHK1 and WEE1) and other key DDR proteins (CDK1, DNA-PKcs, Ku70 and Ku80) were measured by densitometry analysis of western blots. Representative images are of the Western blots and Ponceau staining with protein ladder and loading control protein, α-tubulin as given in main Figure 1A.

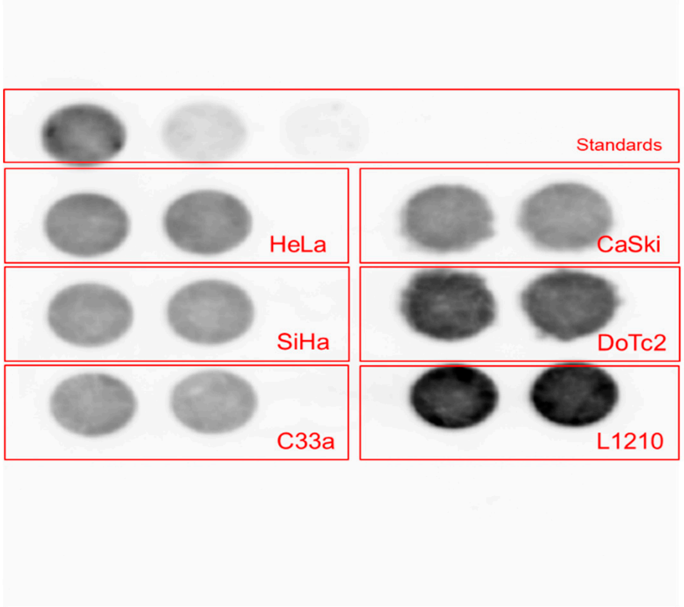

Supplementary Figure S3. Representative immunoblot images of the PARP activity among the cervical cancer cell lines: A GCLP-validated assay was used to measure DNA damage-activated PARP activity in permeabilised cells, in the presence of NAD+ substrate (350 nM) and a 12 mer palindromic double-stranded oligonucleotide (10 mg/ml) (Invitrogen, Waltham, MA, USA) to activate PARP1, by immunological detection of the product (PAR), using 10H Ab (Enzo life sciences, Farmingdale, NY, USA) and secondary HRP-conjugated goat anti-mouse Ab (Dako, Santa Clara, CA, USA), as described previously [1, 2]. Endogenous PAR levels were measured in the absence of activating oligonucleotide and NAD+. PARP activity was measured after the stimulation of the cells in the presence of oligonucleotide and NAD. For the calculation of the endogenous PAR cells were used but without oligonucleotide and NAD. L1210 cell line was used as control.

Cell line	HeLa	SiHa	C33a	CaSki	DoTc2
Mean endogenous PAR	7.2	1.6	2.8	2.2	6.3
Mean PARP activity*	24683	18144	21026	20548	36233
* PARP activity unit is in pmols PAR /10^6cells					

Supplementary Table S3. PARP activity among the cervical cancer cell lines: Endogenous PAR and maximum PARP activity after stimulation of the cervical cancer cells. Numbers are in pmol PAR/10^6cells.

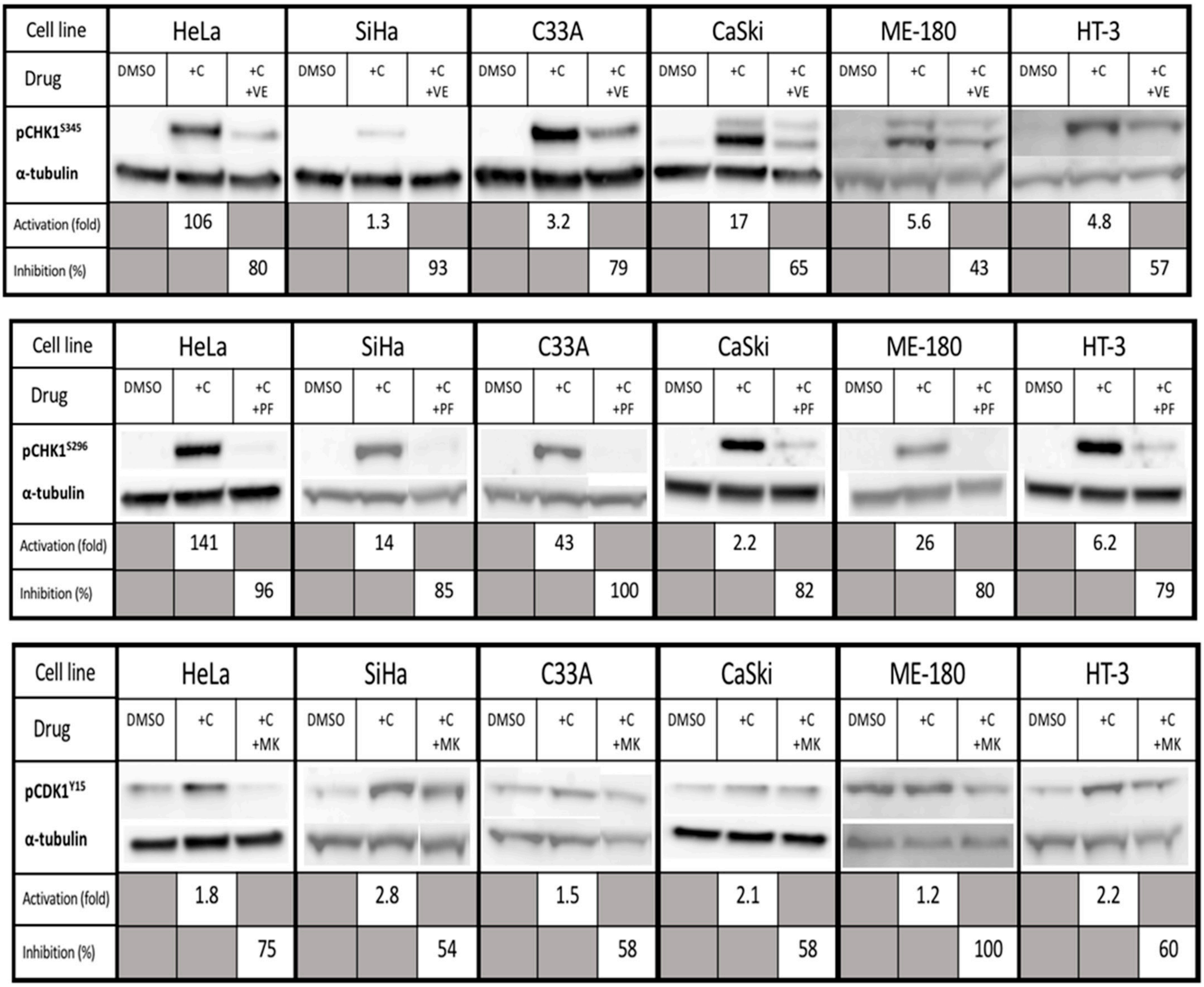

Supplementary Figure S4. DDR pathways activation and inactivation as given in main Figure 2C and 2D: Representative images are of the Western blots of ATR, CHK1 and WEE1 activation in response to cisplatin and inactivation in response to the ATR, CHK1 and WEE1 inhibitors as given in main Figure 2C and 2D.
Exponentially growing cell exposed to 0.5 % DMSO alone, 3 μM cisplatin and 3 μM cisplatin + 1 μM VE-821, 50 nM PF-477736 or 100 nM MK-1775 for 24 hours prior to harvest cells and lysate preparation.
Fold activation of ATR by cisplatin is given as a ratio of the $pCHK1^{S345}$ band intensity to that of the untreated (DMSO) control. % inhibition is the percent-reduction in $pCHK1^{S345}$ band intensity of the Cisplatin + VE-821 treated cells compared to that of cisplatin alone and normalised to the control.
Fold activation of CHK1 by cisplatin is given as a ratio of the $pCHK1^{S296}$ band intensity to that of the untreated (DMSO) control. % inhibition is the percent-reduction in $pCHK1^{S296}$ band intensity of the Cisplatin + PF-477736 treated cells compared to that of cisplatin alone and normalised to the control.
Fold activation of WEE1 by cisplatin is given as a ratio of the $pCDK1^{Y15}$ band intensity to that of the untreated (DMSO) control. % inhibition is the percent reduction in $pCDK1^{Y15}$ band intensity of the Cisplatin + MK-1775 treated cells compared to that of cisplatin alone and normalised to the control.
For any further clarification please check the Stuart Rundle's thesis [41].

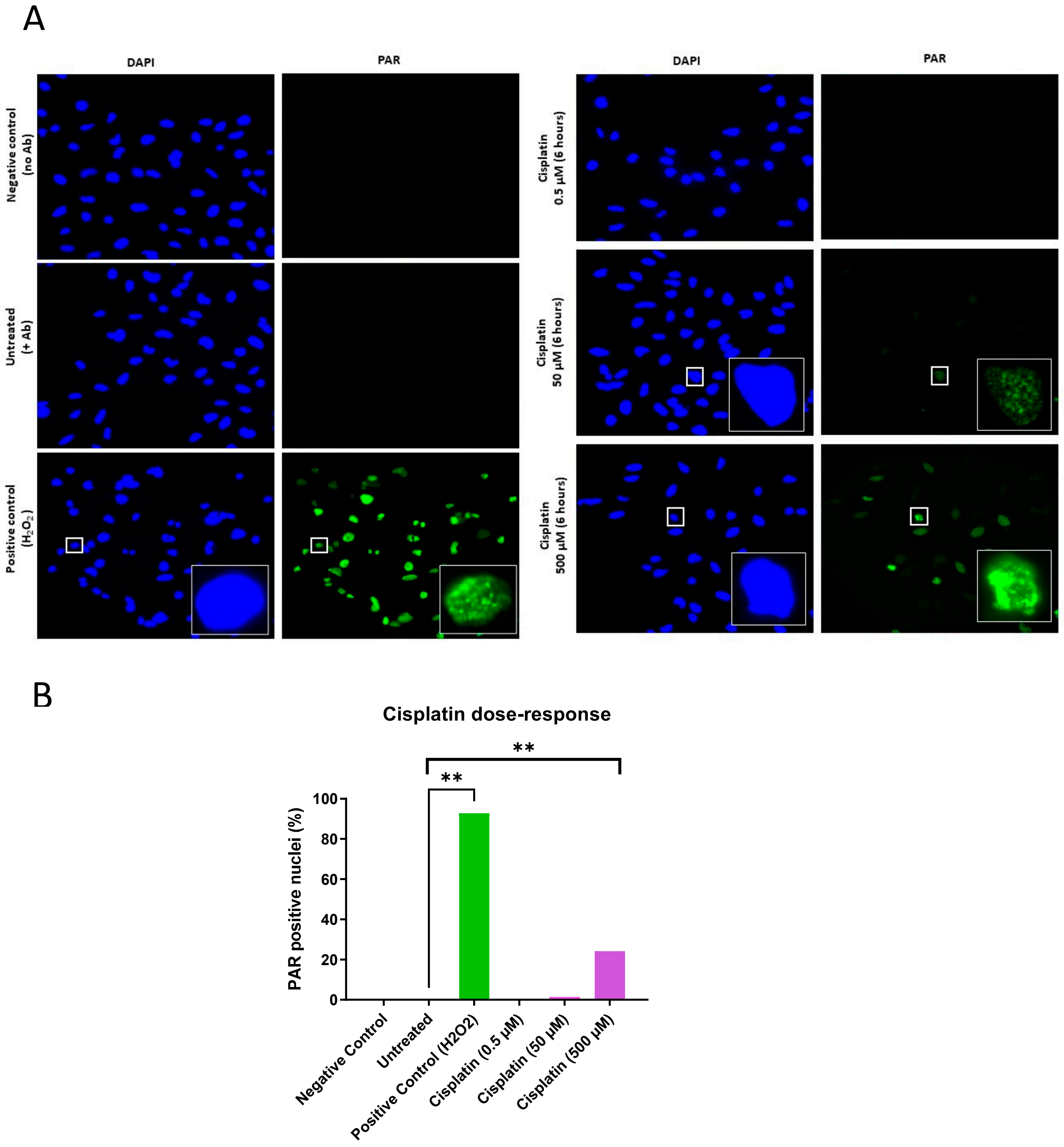

Supplementary Figure S5. Optimisation of cisplatin concentration for PARP activation: It is frequently necessary to use concentrations of a DNA damaging agent that would be supra-lethal in order to see a measurable effect over a short time-frame [4]. Thus, before selecting the cisplatin concentration we optimised the concentration of cisplatin (at 0.5, 50 and 500 µM) that activates PARP using hydrogen peroxide (H2O2), a DNA-damaging agent known to induce PARylation, as a positive control. As cisplatin induces DNA damage less rapidly than H2O2, a 6-hour exposure was chosen based on previous literature [5]. (A) Cisplatin concentration dependent activation of PARP was measured by immunofluorescence detection of the product, PAR, in exponentially growing HeLa cells. Cells were fixed after 6 hr treatment with 0.5, 50 or 500 µM cisplatin at 37 °C. 10 mM H2O2 for 30 mins was used as the positive control (H2O2 concentration optimisation is not shown here). The negative control has no primary antibody. Representative nuclei with PAR activation are shown alongside. (B) A bar chart of the percentage of PAR positive nuclei, data are mean ± SD from four inde-

pendent experiments. During the optimisation stage, we observed that 500 μM cisplatin substantially increase the percentage of PAR positive cells compared to the untreated control (p=0.0085). The 0.5 μM cisplatin and 50 μM cisplatin treatments showed little impact. The use of supra-lethal concentration of cisplatin (300 μM) induced PARP activation measured by immunofluorescence microscopy were previously reported in O-342 rat ovarian tumour cells and CV-l monkey cells [4]. Thus, we have used 500 μM cisplatin only in the experiment as given in Figure 2A.

i

Cell Line	VE-821 LC_{50} μM (± SEM)	PF-477736 LC_{50} nM (± SEM)	MK-1775 LC_{50} nM (± SEM)	Rucaparib LC_{50} μM (± SEM)
Hela	6.5 ± 1.7	168 ± 3.0	275 ± 26	20.8 ± 5.9
SiHa	22.7*	239 ± 20	281 ± 42	12.5 ±4.2
C33A	5.0 ± 2.3	268 ± 41	245 ± 22	11.4 ± 4.0
CaSki	5.8 ± 0.8	88 ± 19	295 ± 93	18.0 ± 3.0
ME-180	15 ± 2.3	323 ± 17	395 ± 105	-
HT-3	5.1 ± 2.3	1328*	716 ± 17	-

ii

	Comparison between the cell lines at highest concentration of the inhibitors
Rucaparib	C33a vs HeLa<br>p= 0.0118 (*)
VE-821	SiHa vs HeLa<br>p=0.0217 (*)
PF-477736	SiHa vs C33a<br>and<br>SiHa vs CaSki<br>p= 0.0753 (NS)
MK-1775	CaSki vs HeLa<br>p=0.0577 (NS)

Supplementary Table S4. The cytotoxicity of the DDR inhibitors alone in the cervical cancer cell lines as given in main Figure 3A-E: (i) LC50 values for each VE-821, PF-477736, MK-1775 and Rucaparib in cervical cancer cell lines as given in main Figure 3A-E. Survival was determined by clonogenic assay. The mean ± Standard error of the mean for three independent experiments is given (*indicates that survival fell to 50% in one-out of three experiments with this cells line and drug). (ii) Comparison between the most sensitive with the least sensitive cells in response to the highest concentration of the inhibitors. Data represents means ± SEM (N=3). The differences between the cells were compared using t-test. * = p < 0.05, NS= non-significant.

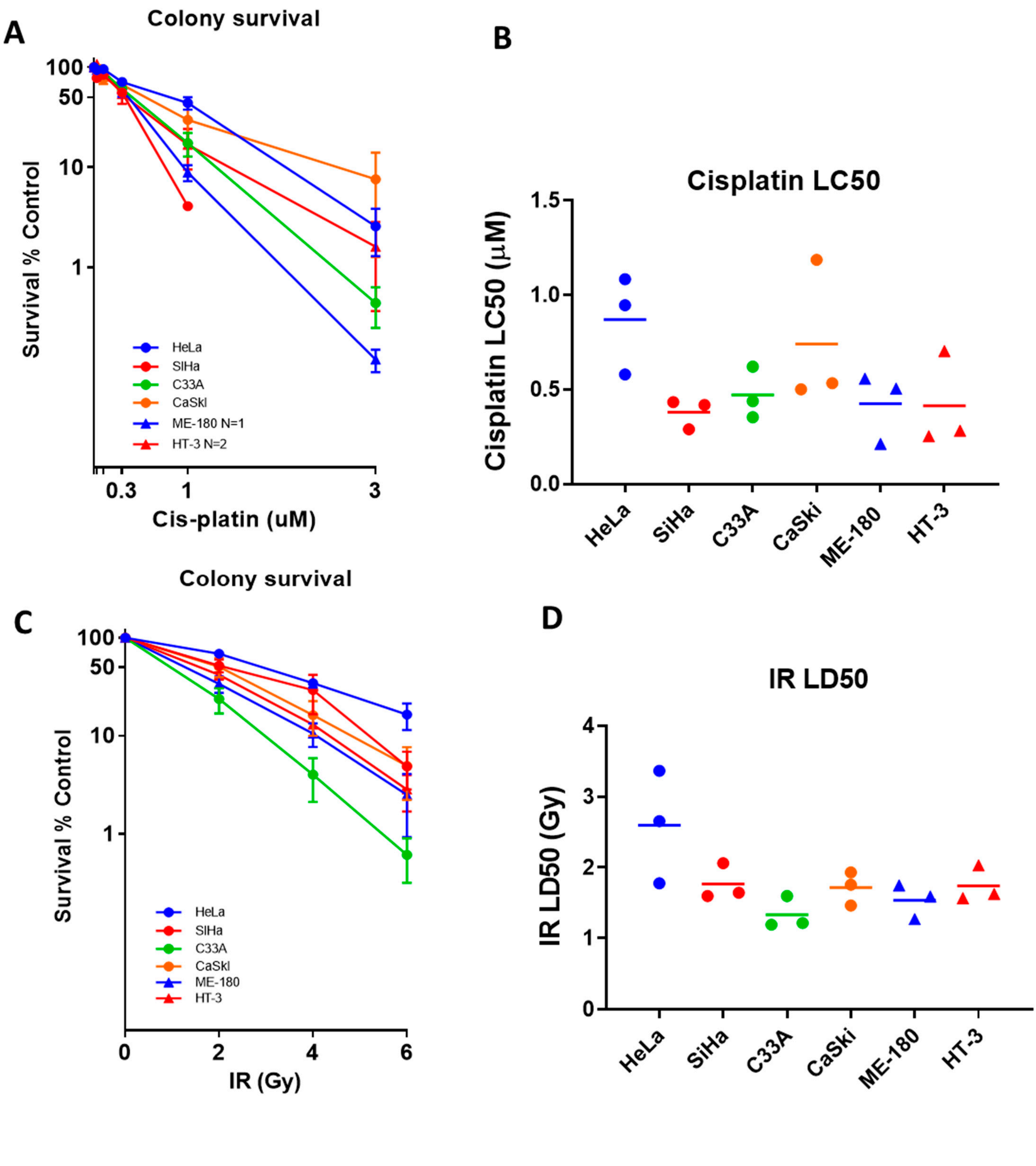

Supplementary Figure S6. Sensitivity to IR and cisplatin to the cervical cancer cells: Survival of cervical cancer cells following exposure to (A) cisplatin and (B) lethal concentration at which 50% cells die i.e. LC50 ; (C) Ionising radiation (IR) and (D) its lethal dose at which 50% cell die i.e. LD50. Survival was determined by clonogenic assay. Survival is given as a percentage relative to the survival in a DMSO only control. Data represents means ± SEM (N=3).

	IR dose at 50% colony survival (LD_{50})			Colony survival at 2 Gy		
Cell Line	IR (Gy ± SEM)	IR + VE-821 (Gy ± SEM)	VE-821 PF_{50} (± SD)	IR (% ± SEM)	IR + VE-821 (% ± SEM)	VE-821 $PF_{2\text{-}IR}$ (± SD)
**HeLa**	**2.6** ± 0.5	**1.5** ± 0.1	**1.7** ± 0.4	**69** ± 5	**38** ± 4	**1.8** ± 0.3 *
**SiHa**	**2.6** ± 0.8	**1.7** ± 0.2	**1.5** ± 0.5	**52** ± 8	**40** ± 5	**1.3** ± 0.1
**C33A**	**1.3** ± 0.1	**1.1** ± 0.1	**1.1** ± 0.2	**24** ± 7	**15** ± 3	**1.6** ± 0.5
**CaSki**	**2.1** ± 0.5	**1.3** ± 0.1	**1.6** ± 0.3	**49** ± 12	**19** ± 8	**2.9** ± 0.7 *
**ME-180**	**1.4** ± 0.2	**1.1** ± 0.0	**1.2** ± 0.2	**34** ± 7	**8** ± 2	**5.1** ± 3.6
**HT-3**	**1.8** ± 0.2	**1.4** ± 0.7	**1.3** ± 1.0	**42** ± 4	**27** ± 4	**1.6** ± 0.3

	IR dose at 50% colony survival (LD_{50})			Colony survival at 2 Gy		
Cell Line	IR (Gy ± SEM)	IR + PF-477736 (Gy ± SEM)	PF-477736 PF_{50} (± SD)	IR (% ± SEM)	IR + PF-477736 (% ± SEM)	PF-477736 $PF_{2\text{-}IR}$ (± SD)
**HeLa**	**2.6** ± 0.5	**2.6** ± 0.1	**1.0** ± 0.3	**69** ± 5	**52** ±1	**1.1** ± 0.3
**SiHa**	**2.6** ± 0.8	**2.1** ± 0.4	**1.2** ± 0.3	**52** ± 8	**47** ± 7	**1.1** ± 0.3
**C33A**	**1.3** ± 0.1	**1.3** ± 0.1	**1.0** ± 0.03	**24** ± 7	**21** ± 6	**1.1** ± 0.05
**CaSki**	**2.1** ± 0.5	**1.8** ± 0.3	**1.2** ± 0.3	**49** ± 12	**39** ± 10	**1.4** ± 0.6
**ME-180**	**1.4** ± 0.2	**1.4** ± 0.2	**1.1** ± 0.1	**34** ± 6	**25** ± 6	**1.5** ± 0.5
**HT-3**	**1.8** ± 0.1	**1.7** ± 0.1	**1.0** ± 0.1	**42** ± 4	**41** ± 5	**1.0** ± 0.2

	IR dose at 50% colony survival (LD_{50})			Colony survival at 2 Gy		
Cell Line	IR (Gy ± SEM)	IR + MK-1775 (Gy ± SEM)	MK-1775 PF_{50} (± SD)	IR (% ± SEM)	IR + MK-1775 (% ± SEM)	MK-1775 $PF_{2\text{-}IR}$ (± SD)
**HeLa**	**2.6** ± 0.5	**2.2** ± 0.4	**1.3** ± 0.6	**70** ± 5	**53** ± 10	**1.3** ± 0.4
**SiHa**	**2.6** ± 0.8	**1.8** ± 0.1	**1.5** ± 0.6	**52** ± 8	**42** ± 3	**1.2** ± 0.2
**C33A**	**1.3** ± 0.1	**1.3** ± 0.1	**1.1** ± 0.1	**24** ± 7	**19** ± 7	**1.5** ± 0.8
**CaSki**	**2.1** ± 0.5	**1.9** ± 0.2	**1.1** ± 0.2	**49** ± 12	**44** ± 6	**1.1** ± 0.2
**ME-180**	**1.4** ± 0.2	**1.3** ± 0.7	**1.2** ± 0.2	**34** ± 6	**25** ± 3	**1.3** ± 0.2
**HT-3**	**1.7** ± 0.1	**2.1** ± 0.2	**0.9** ± 0.2	**42** ± 4	**52** ± 6	**0.8** ± 0.2

	IR Dose at 50 % colony survival (LD50)			Colony survival at 2 Gy		
**Cell line**	**IR (Gy)**	**IR (Gy) + Rucaparib**	**PF_{50}**	**IR(Gy)**	**IR (GY) + Rucaparib**	**PF 2-IR**
	**Mean ± SEM**	**Mean ± SEM**	**Mean ± SEM**	**Mean ± SEM**	**Mean ± SEM**	**Mean**
**HeLa**	4.04±0.38	2.14±0.35	2.03±0.47	73.18 ± 4.05	52.09 ± 7.29	1.4 ± 0.22
**SiHa**	2.46±0.19	1.94±0.15	1.3±0.19	57.44 ± 3.06	48.52 ± 3.12	1.18 ± 0.22
**C33a**	1.85±0.14	1.63±0.06	1.13±0.05	46.27 ±2.88	38.97 ± 2.03	1.18 ± 0.09
**CaSki**	2.58±0.55	1.89±0.22	1.33±0.13	57.80 ± 11.08	46.77 ± 4.22	1.23 ± 0.22

Supplementary Table S5. Radiopotentiation by the DDR inhibitors in the cervical cancer cell lines as given in main Figure 3 F-J: Survival was determined by clonogenic assay. The potentiation factor at 50% survival (PF50) is the ratio between survival at the LD50 for IR alone and the LD50 in combination with DDR inhibitor. Similarly the potentiation factor at fixed dose i.e., 2 Gy ionising radiation (PF2-IR) is the ratio of the survival at these dose in the absence and presence of a DDR inhibitor. The differences between the mean LD50/LD2-IR ± inhibitor was compared using a paired t-test. * = $p < 0.05$

	Concentration at 50% colony survival (LC_{50})			Colony survival at 0.3 µM cisplatin		
Cell Line	Cisplatin (µM ± SEM)	Cisplatin + VE821 (µM ± SEM)	VE-821 PF_{50} (± SD)	Cisplatin (% ± SEM)	Cisplatin + VE821 (% ± SEM)	VE-821 $PF_{0.3\text{-}cis}$ (± SD)
HeLa	0.87 ± 0.15	0.28 ± 0.02	3.1 ± 0.9	71 ± 4	42 ± 4	1.7 ± 0.2 *
SiHa	0.38 ± 0.05	0.21 ± 0.04	1.8 ± 0.2 *	56 ± 4	27 ± 8	2.2 ± 0.8
C33A	0.47 ± 0.08	0.08 ± 0.01	5.9 ± 1.3 *	61 ± 5	12 ± 2	5.0 ± 0.9 **
CaSki	0.74 ± 0.22	0.42 ± 0.12	1.8 ± 0.2	67 ± 3	55 ± 8	1.2 ± 0.2
ME-180	0.42 ± 0.11	0.10 ± 0.02	4.9 ± 3.2	60 ± 10	5 ± 0.2	12.7 ± 2.9 *
HT-3	0.41 ± 0.15	0.19 ± 0.03	2.2 ± 1.1	54 ± 11	35 ± 3	1.6 ± 0.8

	Concentration at 50% colony survival (LC_{50})			Colony survival at 0.3 µM cisplatin		
Cell Line	Cisplatin (µM ± SEM)	Cisplatin + PF-477736 (µM ± SEM)	PF-477736 PF_{50} (± SD)	Cisplatin (% ± SEM)	Cisplatin + PF-477736 (% ± SEM)	PF-477736 $PF_{0.3\text{-}cis}$ (± SD)
HeLa	0.48 ± 0.11	0.48 ± 0.11	1.0 ± 0.1	59 ± 7	65 ± 11	0.9 ± 0.1
SiHa	0.41 ± 0.12	0.35 ± 0.10	1.4 ± 0.01	58 ± 14	45 ± 17	1.9 ± 1.3
C33A	0.41 ± 0.11	0.24 ± 0.01	1.3 ± 0.7	56 ± 12	40 ± 9	1.4 ± 0.5
CaSki	1.04 ± 0.08	0.57 ± 0.07	1.8 ± 0.5	66 ± 4	67 ± 5	1.0 ± 0.2
ME-180	0.42 ± 0.11	0.18 ± 0.04	2.5 ± 1.3	60 ± 10	34 ± 6	1.8 ± 0.3
HT-3	0.41 ± 0.15	0.23 ± 0.06	1.9 ± 1.1	54 ± 11	46 ± 3	1.2 ± 0.5

	Concentration at 50% colony survival (LC_{50})			Colony survival at 0.3 µM cisplatin		
Cell Line	Cisplatin (µM ± SEM)	Cisplatin + MK-1775 (µM ± SEM)	MK-1775 PF_{50} (± SD)	Cisplatin (% ± SEM)	Cisplatin + MK-1775 (% ± SEM)	MK-1775 $PF_{0.3\text{-}cis}$ (± SD)
HeLa	0.56 ± 0.03	0.46 ± 0.08	1.4 ± 0.6	59 ± 7	60 ± 6	1.0 ± 0.3
SiHa	0.41 ± 0.12	0.38 ± 0.10	1.1 ± 0.3	58 ± 14	54 ± 11	1.1 ± 0.3
C33A	0.41 ± 0.11	0.25 ± 0.02	1.7 ± 0.9	56 ± 12	41 ± 4	1.4 ± 0.7
CaSki	1.04 ± 0.80	0.97 ± 0.21	1.1 ± 0.2	66 ± 4	81 ± 10	0.8 ± 0.1
ME-180	0.42 ± 0.11	0.20 ± 0.06	2.3 ± 0.5	60 ± 10	41 ± 4	1.5 ± 0.3
HT-3	0.41 ± 0.15	0.41 ± 0.07	1.0 ± 0.3	54 ± 11	54 ± 5	1.0 ± 0.2

	Concentration at 50 % colony survival (LC 50)			Colony survival at 0.3 uM cisplatin (Cis)		
Cell line	Cisplatin (µM)	Cisplatin (µM) + Rucaparib (µM)	PF_{50}	Cisplatin (µM)	Cisplatin (µM) + Rucaparib (µM)	PF 0.3µM cis
	Mean ± SEM	Mean ± SEM	Mean	Mean ± SEM	Mean ± SEM	Mean
HeLa	0.75 ± 0.08	0.56 ± 0.10	1.33	73 ± 4.50	58 ± 7.02	1.25 ± 0.25
SiHa	0.64 ± 0.10	0.53 ± 0.10	1.2	73.66 ± 8.81	64.33 ± 8.95	1.14 ± 0.04
C33a	0.84 ± 0.05	0.63 ± 0.06	1.33	92.66 ± 10.47	63 ± 3.05	1.47 ± 0.18
CaSki	1.07 ± 0.18	1.13 ± 0.14	0.94	87 ± 7.5	93.66 ± 13.56	0.92 ± 0.40

Supplementary Table S6. Cisplatin sensitisation by the DDR inhibitors in the cervical cancer cell lines as given in main Figure 3K-O: Survival was determined by clonogenic assay. The potentiation factor at 50% survival (PF50) is the ratio between survival at the LC50 for cisplatin alone and the LC50 in combination with DDR inhibitor. Similarly the potentiation factor at fixed concentration i.e., 0.3 µM cisplatin (PF0.3-cis) is the ratio of the survival at these concentrations in the absence and presence of a DDR inhibitor. The differences between the mean LC50/LC0.3-cis ± inhibitor was compared using a paired t-test. * = $p < 0.05$

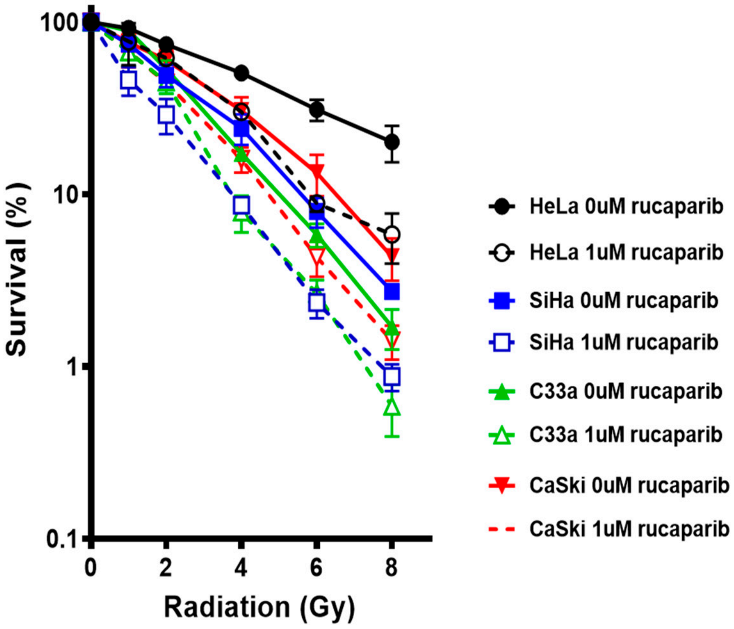

Cell line	LC_{50} Cisplatin + Radiation Only	LC_{50} Cisplatin + Radiation + Rucaparib	PF_{50}	*p-value*
	Mean ± SEM	Mean ± SEM	Mean ± SEM	
**HeLa**	4.07±0.19	2.22±0.72	2.58±1.14	**0.04**
**SiHa**	2.09±0.38	1.07±0.23	2±0.13	**0.01**
**C33a**	2.23±0.24	1.69±0.38	1.42±0.22	**0.03**
**CaSki**	2.65±0.48	1.60±0.26	1.64±0.06	**0.02**

Supplementary Figure S7. Chemo-radiosensitisation by rucaparib in cervical cancer cells: Potentiation of chemo-radiation by rucaparib in cervical cancer cells. Survival of cells following exposure to chemo-radiation alone (continuous line) and in combination with 1 µM rucaparib (dashed line). Cells were exposed to increasing doses of radiation (0-8 Gy) and 1 µM cisplatin, with or without addition of rucaparib, and survival was determined by clonogenic assay. For the chemo-radiation alone data were normalised to DMSO and for combinations they were normalised to cisplatin. Data represents means ± SEM (N=3).

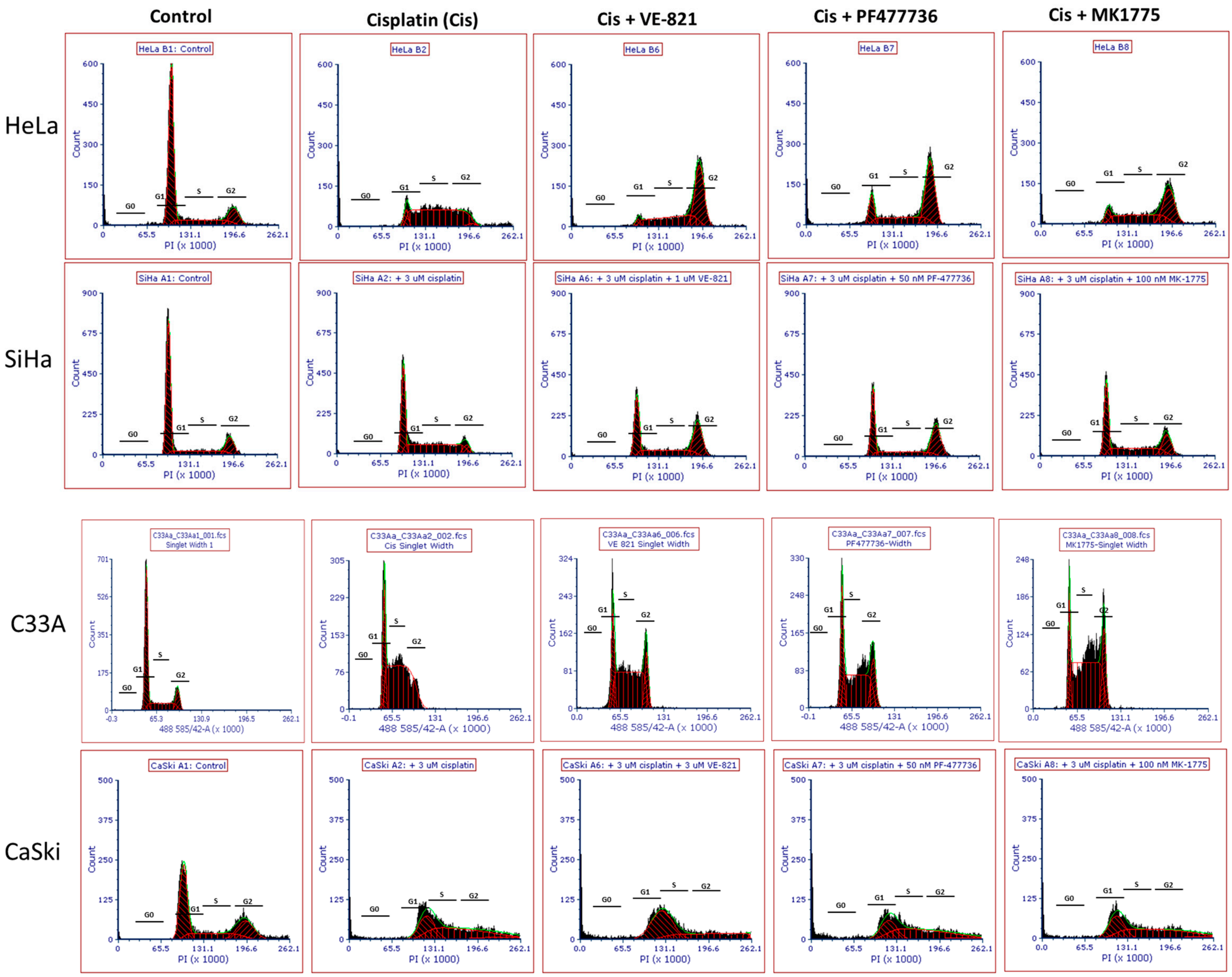

Supplementary Figure S8. Cell cycle histogram profiles of cervical cancer cell lines treated with vehicle control (0.5% DMSO), or 3 µM cisplatin (Cis) +/- 1 µM VE-821 (VE), 50 nM PF-477736 (PF) or 100 nM MK-1775 (MK) as given in Figure 4A and in Supplementary Table S7.

Cell line	inhibitor	Cisplatin	% single cell population							
			Sub-G1		G1		S		G2/M	
			A	B	A	B	A	B	A	B
HeLa	-	-	3.7	3.8	75	69	13	17	8.5	11
	-	+	3.3	2.3	25	19	57	64	15	15
	VE-821	-	4.8	4.0	72	71	15	16	8.4	9.3
	PF-477736	-	5.4	4.1	75	68	12	18	7.1	10
	MK-1775	-	4.6	4.3	72	70	13	19	10	6.4
	VE-821	+	4.0	2.6	12	6.7	34	34	50	56
	PF-477736	+	4.9	3.8	24	17	23	27	48	52
	MK-1775	+	4.9	3.7	20	13	33	36	42	47
SiHa	-	-	0.6	0.3	68	77	17	10	14	13
	-	+	0.3	0.4	47	45	41	45	13	9.2
	VE-821	-	0.5	0.9	74	76	13	13	12	11
	PF-477736	-	0.5	0.6	75	78	12	10	12	11
	MK-1775	-	0.4	0.4	72	77	14	12	14	11
	VE-821	+	0.5	0.5	37	46	24	27	39	27
	PF-477736	+	0.6	0.6	39	53	22	20	38	26
	MK-1775	+	0.4	0.7	42	54	35	31	22	15
C33A	-	-	1.1	0.8	56	59	21	20	22	21
	-	+	0.6	0.8	25	29	56	57	18	14
	VE-821	-	1.4	0.7	57	63	20	17	21	19
	PF-477736	-	1.6	0.7	59	62	21	19	19	19
	MK-1775	-	0.6	1.2	58	61	20	22	22	16
	VE-821	+	1.6	1.3	21	15	46	50	32	34
	PF-477736	+	1.3	1.4	24	26	46	48	29	25
	MK-1775	+	0.6	0.7	16	20	53	51	30	27
CaSki	-	-	1.2	0.5	59	55	20	21	19	23
	-	+	3.7	0.2	15	36	68	53	14	11
	VE-821	-	1.4	0.4	56	55	22	21	21	24
	PF-477736	-	1.9	0.5	56	52	22	24	21	23
	MK-1775	-	1.9	0.6	55	52	19	23	24	24
	VE-821	+	6.1	0.3	30	31	53	60	11	8.8
	PF-477736	+	6.3	0.4	29	32	48	59	17	8.6
	MK-1775	+	6.3	0.4	35	37	43	53	16	10
ME-180	-	-	0.1	0.4	56	71	22	12	21	17
	-	+	0.4	1.5	30	18	60	71	8.9	9.3
	VE-821	-	0.4	0.4	62	77	22	11	16	12
	PF-477736	-	0.4	0.3	60	74	21	11	18	15
	MK-1775	-	0.9	0.5	60	76	21	10	18	13
	VE-821	+	1.1	0.4	7.2	5.4	89	87	3.1	7.0
	PF-477736	+	1.3	0.7	8.5	7.4	86	84	4.6	8.4
	MK-1775	+	1.7	0.5	17	10	77	77	3.7	13
HT-3	-	-	1.8	1.2	66	59	17	18	16	22
	-	+	1.9	2.4	26	23	48	58	24	16
	VE-821	-	1.4	1.0	66	58	15	18	17	23
	PF-477736	-	1.4	1.4	66	58	16	17	17	23
	MK-1775	-	1.8	0.9	67	54	16	18	16	27
	VE-821	+	7.3	4.1	17	18	61	65	15	12
	PF-477736	+	4.5	3.4	21	22	52	60	22	15
	MK-1775	+	4.1	2.1	21	24	51	58	24	17

Supplementary Table S7. Cell cycle profiles of cervical cancer cell lines treated with vehicle control (0.5% DMSO), or 3 μM cisplatin (Cis) +/- 1 μM VE-821 (VE), 50 nM PF-477736 (PF) or 100 nM MK-1775 (MK) as given in Figure 4A.

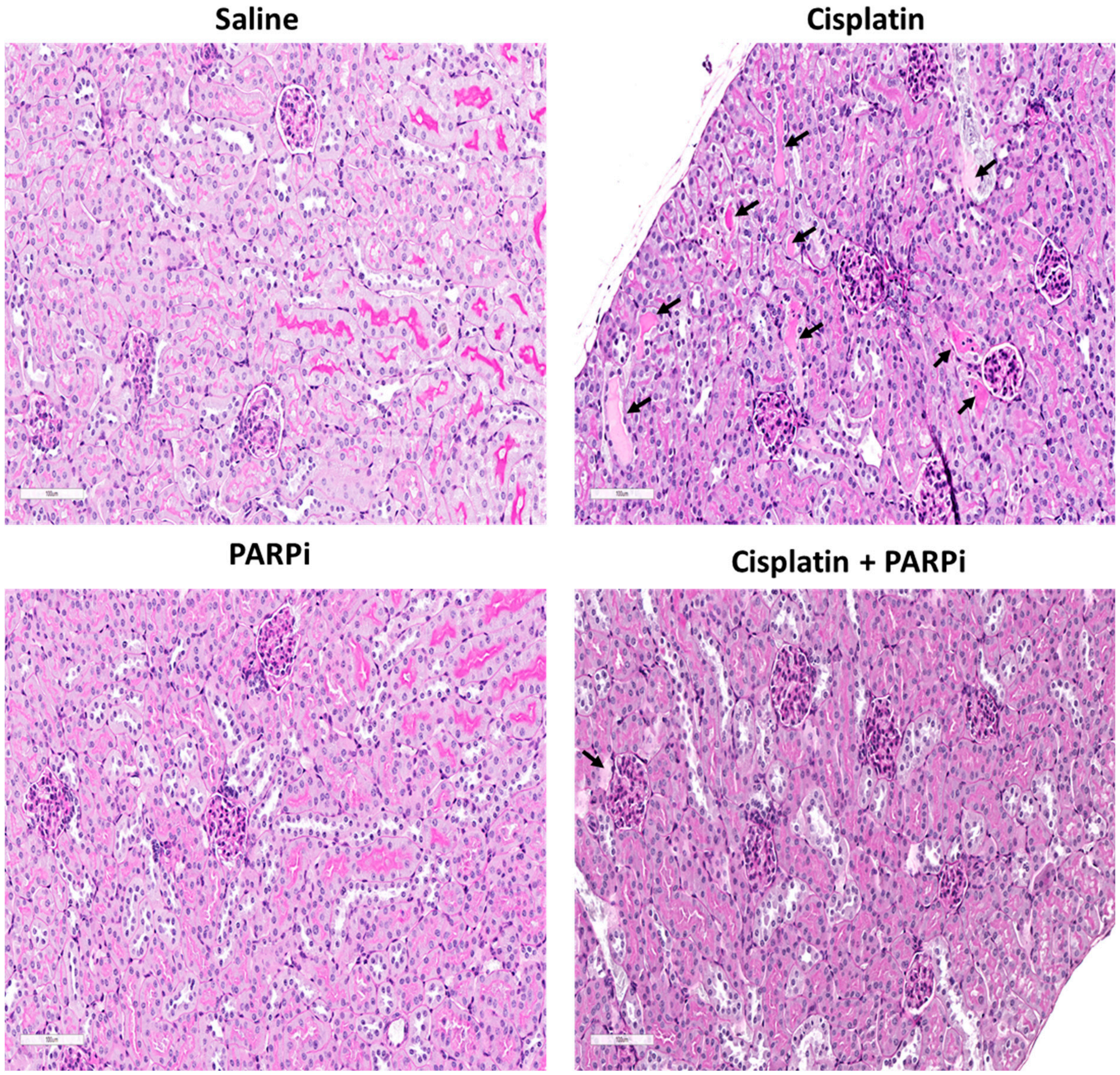

Supplementary Figure S9. Histology of kidney sections: For the histology of the kidney sections, periodic acid schiffs (PAS) staining was done. PAS staining can differentiate cellular morphologies and cellular components (e.g. glycogen, mucopolysaccharides, basement membrane, colloid materials etc.). PAS positive substances stain pink to red and nuclei stain blue due to counterstaining with hematoxylin. For the staining, standard procedure of the PAS kit (Sigma 395B) was followed using the formalin-fixed and paraffin embedded (FFPE) kidney sections. Briefly, 4 μM sections of the kidney on glass slides were deparaffinised and hydrated using deionised water. Slides were then immersed in 'Periodic Acid' solution for 5 minutes at room temperature (18–26°C). This, followed by rinsing the slides in several changes with distilled water. Slides were then immersed in 'Schiff's Reagent' for 15 minutes at room temperature (18–26°C). Next, slides were washed in hot running tap water for 5 minutes. Counterstaining was achieved using hematoxylin solution for 90 seconds. Slides were rinsed again in running tap water. Finally, slides were dehydrated and mounted in mounting media.

Representative images of kidney histology by PAS staining to confirm cisplatin-induced kidney injury by formation of tubular casts at the cortex regions of the kidney sections (indicated with arrows) and rucaparib (PARP inhibitor) in combination with cisplatin shows amelioration of kidney injury by reduced cast formation (indicated with arrow). Histology slides were scanned under the Aperio image scanner, 20X magnification. Scale bar 50 μM.

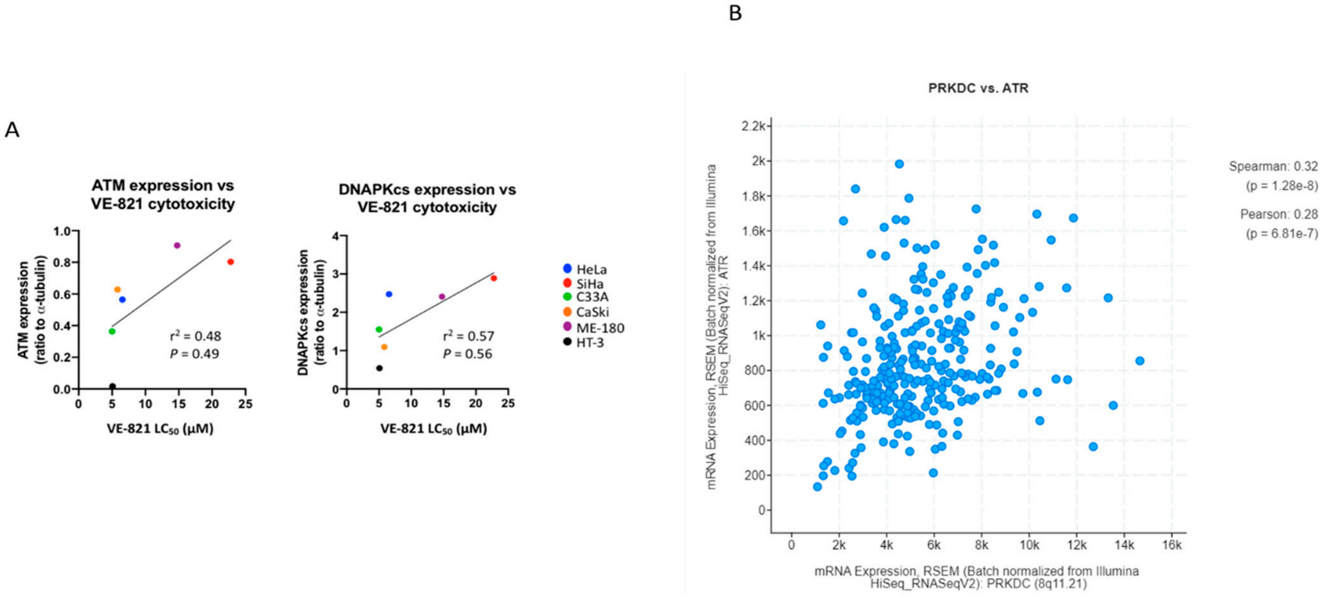

Supplementary Figure S10. Determinants of sensitivity to the DDR inhibitors: (A) Corelation analysis of DDR proteins ATM and DNA-PKcs in response to ATR inhibitor VE-821 in cervical cancer cell lines. Data are taken from Figure 1B, 3B, 3E and supplementary table S4. (B) Data are from publicly available (cBioPortal) TCGA cervical cancer dataset of co-relation between ATR and PRKDC (DNA-PKcs).

Supplementary references:

50. Plummer, R.; Jones, C.; Middleton, M.; Wilson, R.; Evans, J.; Olsen, A.; Curtin, N.; Boddy, A.; McHugh, P.; Newell, D.; Harris, A.; Johnson, P.; Steinfeldt, H.; Dewji, R.; Wang, D.; Robson, L.; Calvert, H. Phase I study of the poly(ADP-ribose) polymerase inhibitor, AG014699, in combination with temozolomide in patients with advanced solid tumors.Clin Cancer Res. 2008,14, 7917-23.

51. Drew, Y.; Ledermann, J.; Hall, G.; Rea, D.; Glasspool, R.; Highley, M.; Jayson, G.; Sludden, J.; Murray, J.; Jamieson, D.; Halford, S.; Acton, G.; Backholer, Z.; Mangano, R.; Boddy, A., Curtin, N.; Plummer, R. Phase 2 multicentre trial investigating intermittent and continuous dosing schedules of the poly(ADP-ribose) polymerase inhibitor rucaparib in germline BRCA mutation carriers with advanced ovarian and breast cancer. Br J Cancer. 2016,114, e21.

52. Bürkle, A.; Chen, G.; Küpper, J. H.; Grube, K.; Zeller, W. J. Increased poly(ADP-ribosyl)ation in intact cells by cisplatin treatment. Carcinogenesis. 1993, 14, 559–561.

53. Gunn, A. R.; Banos-Pinero, B.; Paschke, P.; Sanchez-Pulido, L.; Ariza, A.; Day, J.; Emrich, M.; Leys, D.; Ponting, C. P.; Ahel, I.; Lakin, N. D. The role of ADP-ribosylation in regulating DNA interstrand crosslink repair. Journal of cell science. 2016, 129, 3845–3858.
